# Supplementary material for: Phytochemicals and Antioxidant Capacities of Young Citrus Fruits Cultivated in China
Source: Molecules. 2022 Aug 15;27(16):5185. doi: 10.3390/molecules27165185 (PMC9414917; doi:10.3390/molecules27165185)
Supplement: Supplementary file 1 [file molecules-27-05185-s001.zip › molecules-1858986-supplementary.pdf]

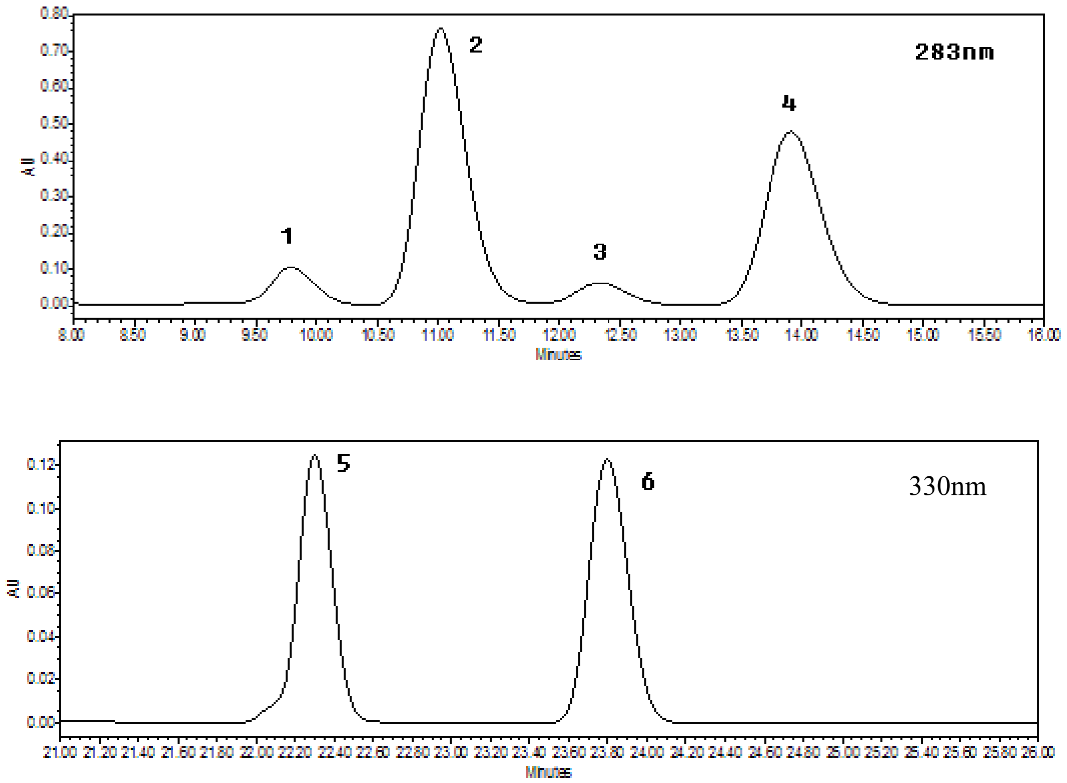


**Figure S1.** HPLC profiles of flavanone glycosides and PMFs standards at 283nm and 330nm
(1: narirutin 2: naringin 3: hesperidin 4: neohesperidin 5: nobiletin 6: tangeretin).

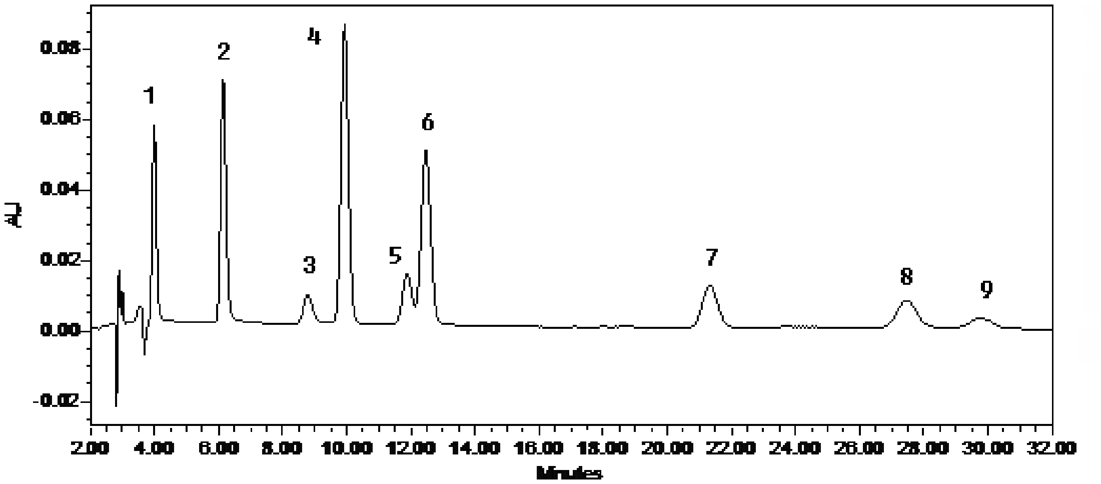


**Figure S2.** HPLC profiles of phenolic acid standards at 280nm
(1: gallic 2: protochatechuic 3: chlorogenic 4: *p*-hydroxybenzoic 5: caffeic 6: vanillic 7: *p*-coumaric 8: ferulic 9: sinapic)

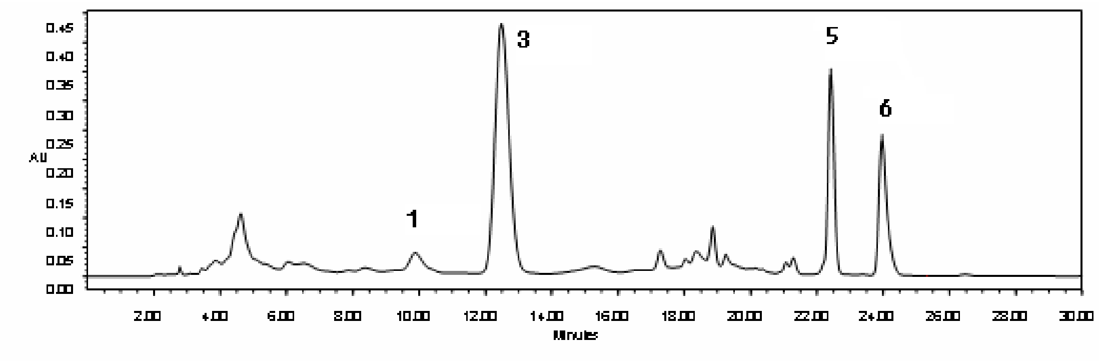


Ponkan

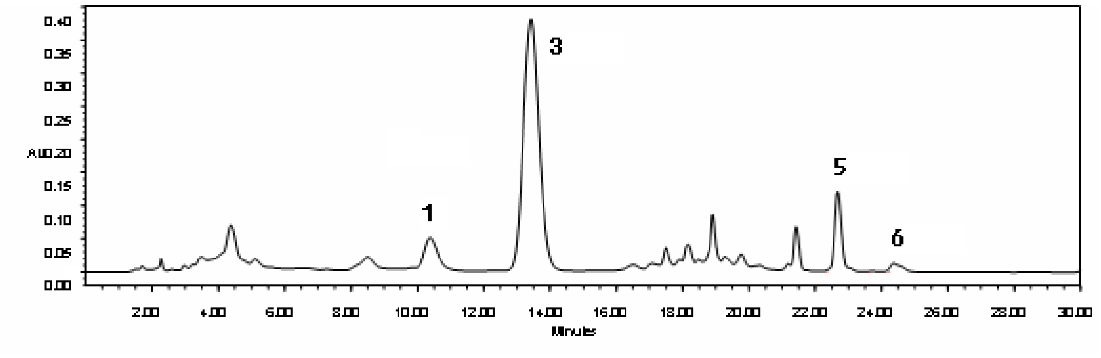


Tiancheng

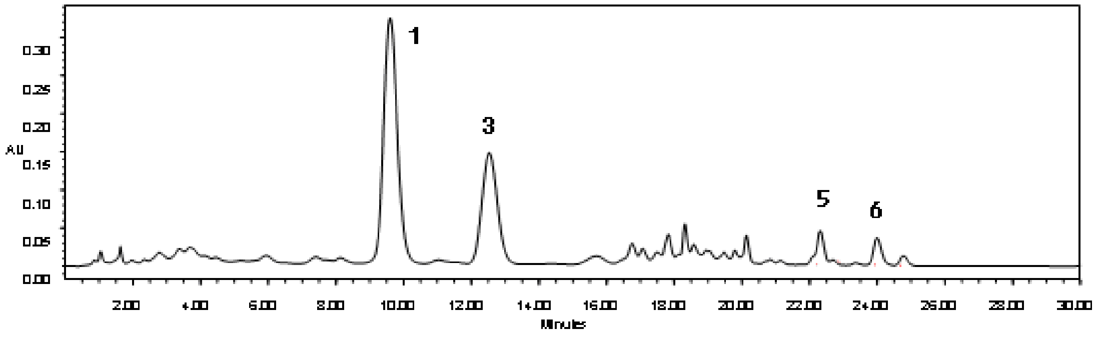


Zaoshuwenzhoumigan

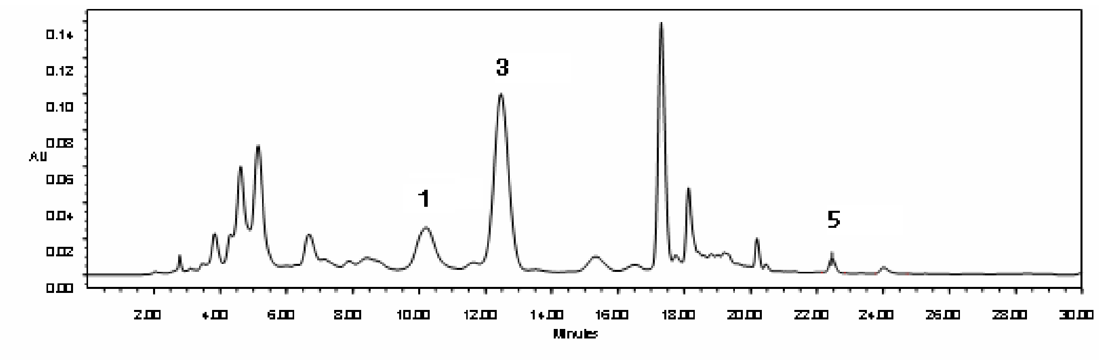


Wanshuwenzhoumigan

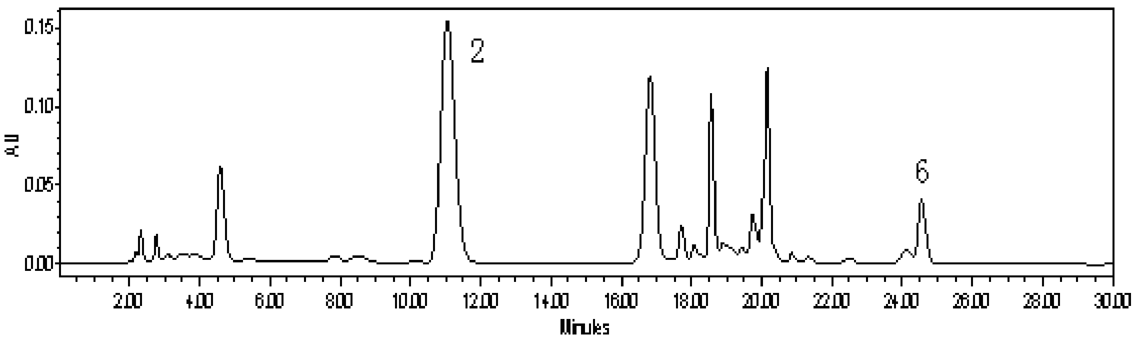


Ougan

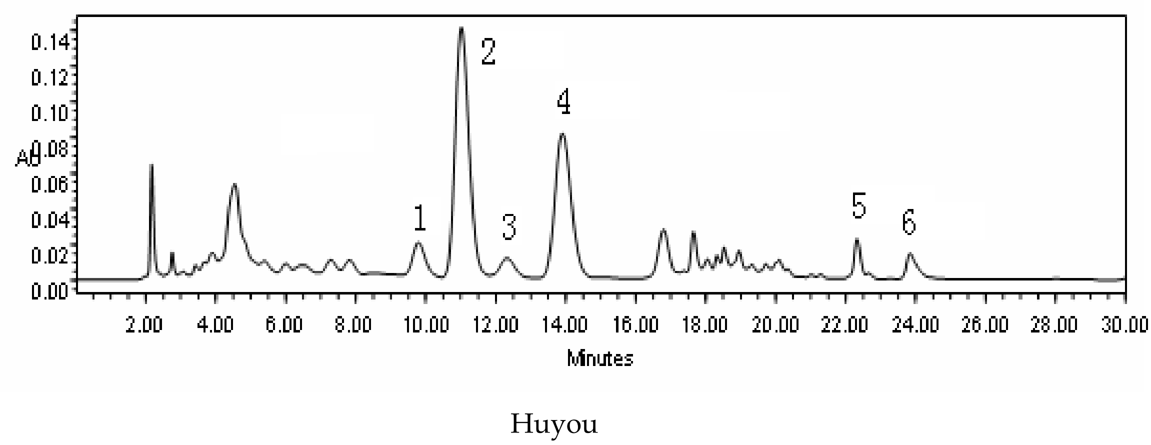


Huyou

**Figure S3.** HPLC profile of the PGs and PMFs of the sample at 330nm
(1: narirutin 2: naringin 3: hesperidin 4: neohesperidin 5: nobiletin 6: tangeretin)
